# Tumor Microenvironment and Immune Response in Lip Cancer

**DOI:** 10.3390/cancers15051478

**Published:** 2023-02-25

**Authors:** Anastasia G. Gkegka, Michael I. Koukourakis, Maria Lambropoulou, Alexandra Giatromanolaki

**Affiliations:** 1 Department of Pathology, Democritus University of Thrace, University Hospital of Alexandroupolis, 68100 Alexandroupolis, Greece; 2 Department of Radiotherapy/Oncology, Democritus University of Thrace, University Hospital of Alexandroupolis, 68100 Alexandroupolis, Greece; 3 Laboratory of Histology—Embryology, Democritus University of Thrace, University Hospital of Alexandroupolis, 68100 Alexandroupolis, Greece

**Keywords:** lip cancer, tumor infiltrating lymphocytes, tertiary lymphoid structures, hypoxia, angiogenesis, FOXP3

## Abstract

**Simple Summary:**

The presence of tumor-infiltrating lymphocytes (TILs) in the tumor milieu has been linked to better patient prognosis and response to chemo-radiotherapy in head and neck carcinoma, among other malignancies. We studied 60 cases of squamous cell carcinoma of the lip to assess the presence of TILs and their subpopulations and tertiary lymphoid structures (TLSs) in the invading front and inner stroma of the tumor. Thereafter, we investigated the impact of histopathological parameters and prevailing conditions of the tumor microenvironment (TME) in TIL and TLS density. Lip SCCs appeared as more immunogenic compared to other HNCs. Poor lymphocytic infiltration was related to larger tumors, higher invasive ability, dense presence of activated stroma fibroblasts and expression of hypoxia markers. High angiogenic activity was linked with high CD4^+^, FOXP3^+^, and low CD8^+^ TIL density, as well as high CD68^+^ macrophage presence. LDH5 expression was linked with high CD4^+^ and FOXP3^+^ TIL density.

**Abstract:**

Tumor-infiltrating lymphocytes (TILs) play a significant role in cancer progression and prognosis of patients. The tumor microenvironment (TME) may affect the anti-tumor immune response. We examined the TIL and tertiary lymphoid structure (TLS) density in the invading front and inner tumor stroma, and the lymphocyte subpopulation (CD8, CD4, FOXP3) density in 60 squamous cell carcinomas of the lip. Analysis was performed in parallel with markers of hypoxia (hypoxia-inducible factor (HIF1α), lactate dehydrogenase (LDHA)) and angiogenesis. Low TIL density in the invading tumor front was related with larger tumor size (*p* = 0.05), deep invasion (*p* = 0.01), high smooth-muscle actin (SMA) expression (*p* = 0.01), and high HIF1α and LDH5 expression (*p* = 0.04). FOXP3^+^ TILs infiltration and FOXP3^+^/CD8^+^ ratios were higher in inner tumor areas, linked with LDH5 expression, and higher MIB1 proliferation index (*p* = 0.03) and SMA expression (*p* = 0.001). Dense CD4^+^ lymphocytic infiltration in the invading front is related to high tumor-budding (TB) (*p* = 0.04) and angiogenesis (*p* = 0.04 and *p* = 0.006, respectively). Low CD8^+^ TIL density, high CD20^+^ B-cell density, high FOXP3^+^/CD8^+^ ratio and high CD68^+^ macrophage presence characterized tumors with local invasion (*p* = 0.02, 0.01, 0.02 and 0.006, respectively). High angiogenic activity was linked with high CD4^+^, FOXP3^+^, and low CD8^+^ TIL density (*p* = 0.05, 0.01 and 0.01, respectively), as well as high CD68^+^ macrophage presence (*p* = 0.003). LDH5 expression was linked with high CD4^+^ and FOXP3^+^ TIL density (*p* = 0.05 and 0.01, respectively). Further research is needed to explore the prognostic and therapeutic value of TME/TIL interactions.

## 1. Introduction

Lip cancer is the second most common skin cancer of the head and neck area [1,2], and the lip is considered the most frequent site of cancer development in the oral cavity [3,4]. In the majority of cases, the lower lip is affected, and men suffer five to eight times more often than women [5,6]. Sun exposure, tobacco use, and alcohol consumption are among the most common causes [7,8], and most patients are diagnosed with squamous cell carcinoma (90%). Therapeutic treatment includes surgery, radiotherapy, and chemotherapy [9]. The five-year survival, however, remains at about 50% [10,11,12], and patients may acquire aesthetic or functional problems after an extensive surgical procedure. In recent years, the focus has been placed on the tumor microenvironment and especially on immune cells and their interaction with cancer cells. Immunotherapy with monoclonal antibodies against PD-1/PD-L1 has been approved for the treatment of squamous cell head–neck cancer [13].

Intense infiltration of tumors with lymphocytes (TILs) has been associated with good prognosis and better response to chemo-radiotherapy in a variety of malignancies, including head–neck cancer [14,15,16,17]. Cancer cells create an immunosuppressive surrounding in order to escape immunity and metastasize [18,19]. The tumor microenvironment (TME) is often characterized by low oxygen presence and pH as a result of impaired vasculature and blood flow, intense glycolysis, and anaerobic cancer cell metabolism [20]. Such physicochemical conditions may directly affect the ability of immune cells to enter, survive and exert their cytotoxic activity in the tumor. Indeed, hypoxia and acidity have been directly linked with compromised proliferation ability of lymphocytes and reduced cytotoxic efficacy of T-cells and NK cells [21,22,23]. Moreover, in recent studies, we showed that tumor hypoxia and anaerobic metabolism are directly linked with an immunologically cold microenvironment and poor prognosis in lung and breast cancer [24,25].

Studying the role of TME conditions in the immune anti-tumor response may provide important insights to help the individualization of tumor immunotherapy. In the current study, we investigated the density of TILs and of tertiary lymphoid structures (TLSs) in the invading front of the tumor and inside the tumor stroma, in a series of surgical specimens from patients with squamous cell carcinoma (SCC) of the lip. These immune features were examined in association with histopathological parameters, such as tumor size, invasiveness, tumor budding, markers of hypoxia and anaerobic metabolism, and other characteristics including cell proliferation, p16, and SMA expression. Lymphocyte subset specific markers of cytotoxic and regulatory T-cells were also examined in parallel with the TME-related markers.

## 2. Materials and Methods

Surgical samples from sixty patients with lip SCC treated with wide or V-shaped surgical excision were included in the study. Cases were serial in time of surgical sample registration in the archives of our Pathology Department. Representative sections were obtained from each specimen, and formalin-fixed paraffin-embedded (FFPE) blocks of tissue were created. In addition, tissue samples from a series of 33 laryngeal SCCs and 13 cases of SCC of the tongue were analyzed to assess differences in the density of lymphocytic infiltration. The study has been approved by the local Ethics and Research Committee of the University Hospital of Alexandroupolis (ES11/26-11-2018).

The histological parameters examined in the present study were the tumor size (in cm), the depth of invasion (DOI) of the tumor (in mm) and tumor budding. TIL and TLS density were also compared to those of a series of 33 laryngeal SCCs and a series of 13 SCCs of the tongue.

### 2.1. Assessment of TILs

TIL evaluation was performed in hematoxylin and eosin-stained slides of one tissue section selected among the sections obtained from all available formalin-fixed paraffin-embedded blocks. Selected slides had a clear invading tumor front and sufficient overall material with evident stroma and lack of extensive necrosis. All high-power ×40 fields were studied in the whole slide, and the number of TILs was estimated separately in all optical fields in the invading front and the inner stroma of the tumor. ‘Invading front’ (or ‘tumor periphery’) was defined as the most peripheral ×40 optical field of the invading front of the tumor, containing both the tumor invading front and normal tissue beside this front (50% normal and 50% cancer). Areas of necrosis or ulceration were excluded. The total number of lymphocytes was divided by the overall number of optical fields (o.f.) on the analogous slide to provide the final score of TIL density for each case (mean value of all fields) in the tumor periphery and in the inner areas. The median value of the acquired scores was used to categorize tumors into two groups of low- and high-TIL density.

### 2.2. Assessment of TLSs

All tertiary lymphoid structures with or without distinguishable germinal centers were counted in hematoxylin and eosin-stained slides. The number of TLSs was evaluated in all high-power ×40 fields in the invading front of the tumor and the inner tumor stroma, separately. Areas of necrosis or ulceration were excluded. The amount of all TLSs was divided by the whole number of optical fields (o.f.) on the corresponding slide, and a final score was acquired for each case (mean value of all fields), in the invading front and inner areas separately. Tumors were grouped into two categories of low- and high-TLS density according to the median value of the obtained scores.

### 2.3. Assessment of Tumor-Budding (TB)

Tumor budding (TB) was assessed according to the recommendations of the International Tumor Budding Consensus Conference (ITBCC) 2016 [26]. Tumor-budding was defined as any cluster of one to four cancer cells in the invading front of the tumor, that had no link with it. The entire tissue slide was examined in a low-power field (magnification, ×40 and ×10), the area with the higher density in TB (hot-spot) was selected, and the tumor buds were estimated in one ×20 optical field. Using the median value (≤median vs. >median) cases were categorized into low and high TB groups.

### 2.4. Immunohistochemistry

Immunohistochemistry for markers (except LDH5) was executed as follows: Sections, 3-μm thick, were deparaffinized and placed in antigen retrieval target solution pH 9.0 (DAKO), followed by microwaving (3 × 5 min). The primary monoclonal antibodies were applied. Following washing with phosphate-buffered saline (PBS), endogenous peroxidase was quenched with EnVision Flex Peroxidase Block (DAKO) for 10 min, then sections were washed with PBS. Non-specific binding was blocked in EnVision Flex mouse Linker for 15 min (DAKO), and then sections were washed with PBS. Sections were then incubated with a secondary antibody (EnVision Flex/HRP; DAKO) for 30 min, and washed in PBS. The color was developed by 5 min incubation with 3,3′-diaminobenzidine (DAB) solution (for CD31 marker was also used HRP Magenta Substrate Chromogen System/DAKO), and sections were counterstained weakly with hematoxylin.

The primary antibodies used are as follows:

- SMA: Mouse monoclonal, 1A4 (DAKO), dilution 1:50, 60 min incubation
- Ki-67: Mouse monoclonal, MIB1 (DAKO), dilution 1:60, 60 min incubation
- p16: Mouse monoclonal, IHC016 (GenomeMe), dilution 1:100, 60 min incubation
- HIF1α: Mouse monoclonal, ESEE122 (OXFORD), dilution 1:10, overnight incubation
- LDH5: Mouse monoclonal, clone Ab9002 (Abcam, UK), dilution 1:200, overnight incubation
- CD4: Mouse monoclonal, 4B12 (DAKO), dilution 1:40, 60 min incubation
- CD8: Mouse monoclonal, C8/144B (DAKO), ready to use, 60 min incubation
- FOXP3: Mouse monoclonal, 236A/37 (OXFORD), dilution 1:50, overnight incubation
- CD20: Mouse monoclonal, clone L26, (DAKO), dilution 1:200, 60 min incubation
- CD68: Mouse monoclonal, clone KP1 (Immunologic), dilution 1:300, 60 min incubation
- CD31: Mouse monoclonal, clone JC70 (DAKO), dilution 1:20, 30 min incubation
- VEGF: Mouse monoclonal, clone VG1 (Diagnostic Biosystems), dilution 1:30, 60 min incubation

All incubations with the primary antibodies were performed at room temperature.

A different procedure was followed for LDH5 immunohistochemical marker, as reported in a previous study of ours [27].

Supplemental Figure S1 shows typical immunohistochemical images of SMA, Ki-67, p16, CD4, CD8, FOXP3, CD20, CD68 and VEGF expression (magnification ×20).

### 2.5. Assessment of Immunohistochemical Markers

SMA immunohistochemical marker was used for the assessment of cancer-associated fibroblast (CAF) density. Vessels were used as an internal control (and were excluded from the evaluation). The percentage of positive SMA stroma expression was estimated in all ×20 optical fields, and the mean value was acquired to score each case. Using the median value of these scores, tumors were classified into three groups: low <5%, medium 5–50%, and high >50%.

Assessment of p16 was performed in all high-power ×40 optical fields of the tissue section, and their mean value was used to score each case. Positivity for p16 marker was defined as strong and diffuse cytoplasmic and/or nuclear staining. Given the low expression of p16 in lip cancer, evaluation of the % of p16-expressing cells was performed at higher magnification to guarantee a more accurate measure.

MIB-1 nuclear expression was examined in ×20 optical fields of the tumor in the whole tissue section. The percentage of cells with nuclear MIB1 expression was estimated per field and their mean value was used to score each case.

The patterns of expression of HIF1α and LDH5 that were considered for analysis were strong cytoplasmic and nuclear, as previously reported [24]. The percentage of cancer cells showing these patterns was examined in all ×20 optical fields, and the mean value was used to score each case. Weak cytoplasmic expression was considered as negative. Cases were grouped in two categories of low vs. high HIF1α or LDH5 expression. High expression was defined when cytoplasmic strong expression concerned above 50% of cancer cells and/or when the nuclear expression was higher than 10%. VEGF expression was assessed in the cytoplasm, and the percentage of cancer cells expressing strong staining was recorded in all optical fields. The median value was used to score each case.

Calculation of the number of microvessels was used to rate angiogenesis. Each tumor slide was firstly scanned at low-power fields (magnification, ×4 and ×10). The area with the highest vascular density in the invading front of the tumor was selected and CD31 immunohistochemical marker was used for the examination in the ×20 optical field. Moving toward the tumor core, the immediately adjacent two ×20 optical fields were evaluated. Thus, a total of three fields (t1, t2, and t3) were included in the assessment, from the periphery to the center of the tumor, and three mean values were acquired for each case. Using the ratio of VD t2/t1 and t3/t1, we estimated the vascular survival ability (VSA) of each tumor. This index shows the ability of vessels to survive in the inner tumor area. The method has been considerably verified in previous studies of ours [28].

All three anti-CD20, CD4, CD8, and FOXP3 antibody staining lymphocytes were assessed in the same way. The percentage of CD20^+,^ CD4^+^, CD8^+^, and FOXP3^+^ positive lymphocytes (among the total recognized lymphocytes) was noted in all high-power ×40 optical fields, separately in the invading front, and inside the tumor stroma. As the sum of CD20^+^ B-cells, and CD4^+^ and CD8^+^ T-cells consist of the majority of lymphocyte subtypes, stained sections were assessed in parallel (per case) to define the percentage of stained (for each marker) lymphocytes. Normalization followed to score each case, assuming that the sum produces 100%. A mean value of all fields was obtained for each marker on each case. The regulatory to effector T-cell CD4^+^/CD8^+^ and FOXP3^+^/CD8^+^ (regulatory/effector) ratios were calculated thereafter. Regarding CD68 staining, the stroma area covered by intense presence of CD68^+^ macrophages was quantified in all available optical fields (separately in the invading tumor front and the inner tumor areas), and the median value was used to score cases.

### 2.6. Statistical Analysis

Statistical analysis and the creation of graphs was performed using the GraphPad Prism 7.0 package. The non-parametric Mann–Whitney test (for two variables) or the Kruskal–Wallis non-parametric test with subsequent Dunn test for intergroup comparison (for multiple variables) was used to compare categorical continuous tumor variables. The Wilcoxon matched-pairs signed rank test was used to compare continuous paired variables. A *p*-value < 0.05 was used for significance.

## 3. Results

### 3.1. TIL and TLS Density Is Higher in Lip Cancer Compared to Other HNCs

**Figure 1:**
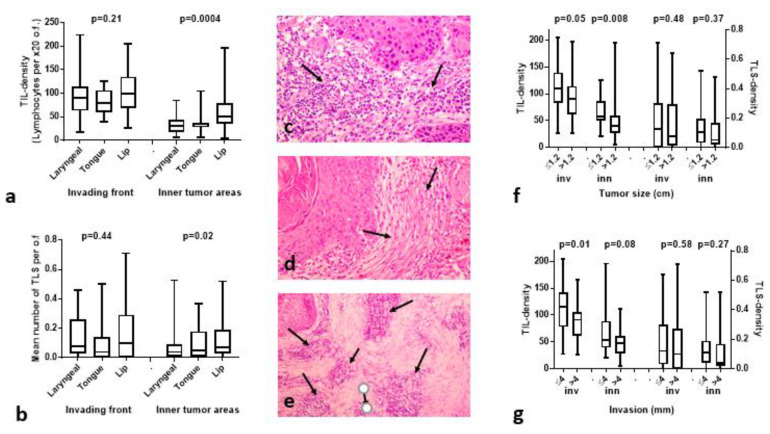
TIL, TLS densities, and histopathological variables. Box and whiskers show the median, range and 25th/75th percentiles: (**a**) comparison of TIL density in the invading front and inner tumor areas according to tumor location; (**b**) comparison of TLS density in the invading front and inner tumor areas according to tumor location; (**c**) high TIL density in the invading tumor front of a lip carcinoma in a H&E tissue section (arrows) at ×20 magnification; (**d**) low TIL density in the invading tumor front of a lip carcinoma in a H&E tissue section (arrows) at ×20 magnification; (**e**) a typical image of a TLSs in the invading front of a lip carcinoma in a H&E tissue section at ×20 magnification; (**f**) TIL and TLS density according to the tumor size, in the invading front (inv) and inner tumor areas (inn); (**g**) TIL and TLS density according to the depth of cancer invasion, in the invading front (inv) and inner tumor areas (inn).

The TIL and TLS densities were separately analyzed and compared for each tumor type (series of 33 laryngeal SCCs, 13 cases of SCC of the tongue, and 60 cases of SCC of the lip). The median TIL density in the tumor periphery was 91, 80 and 99 in laryngeal, tongue and lip carcinomas, respectively (*p* = 0.21). In inner tumor areas, however, TIL density was significantly higher in lip carcinomas compared to other tumors (median 51 vs. 30 and 34 in laryngeal and tongue cancer; *p* = 0.0004). Figure 1a shows the distribution of TILs.

The median TLS density (TLS per o.f.) in the tumor periphery was 0.07, 0.04 and 0.10 in laryngeal, tongue and lip carcinomas (*p* = 0.44). In inner tumor areas, TLS density was significantly higher in lip carcinomas (median 0.07) vs. 0.04 and 0.05 in laryngeal and tongue tumors (*p* = 0.02). Figure 1b shows the distribution of TLSs.

Figure 1c,d shows hematoxylin and eosin sections of two cases with high and low TIL density, respectively, in the invading tumor front. Figure 1e shows tertiary lymphoid structures in hematoxylin and eosin section in the invading tumor front of a lip carcinoma. 

### 3.2. Histopathological Variables vs. TILs/TLSs

The tumor size ranged from 0.5–4.5 (median 1.2) cm. Using the median values, cases were split into two groups of small vs. large tumors (≤median vs. >median). Analysis of TIL density and TLS density in the invading front (inv) and inner tumor areas (inn), showed statistically significant lower TIL values in larger tumors, both in the invading front and inner areas (*p* = 0.05 and 0.008, respectively); Figure 1f. shows that no difference was recorded between TLS densities.

The invasiveness of lip carcinomas was graded by the mm of invasion into the muscle. This ranged from 1.5–17 mm (median 4 mm). Tumors were grouped according to the median (≤median vs. >median). TIL density in the invading front was significantly lower in tumors with deep invasion (median 116 vs. 91; *p* = 0.01), however, there was no difference between TLS scores (Figure 1g).

In lip cancer cases, there was no association of TB numbers with TIL or TLS density. High SMA was linked with low TIL density in the invading tumor front (median TIL density 105 vs. 52; *p* = 0.01).

### 3.3. HIF1α, LDH5 vs. TILs/TLSs

**Figure 2:**
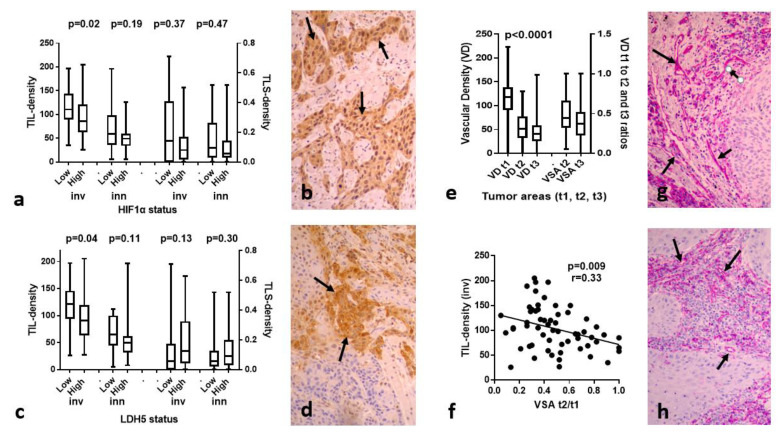
TIL, TLS densities, and hypoxia/angiogenesis variables. Box and whiskers show the median, range and 25th/75th percentiles: (**a**) TIL and TLS density in the invading front and inner tumor areas according to HIF1α expression status; (**b**) Typical immunohistochemical image of a lip carcinoma with high nuclear and cytoplasmic HIF1α expression (arrows); (**c**) TIL and TLS density in the invading front and inner tumor areas according to LDH5 expression status; (**d**) Typical immunohistochemical image of a lip carcinoma with high nuclear and cytoplasmic LDH5 expression (arrows); (**e**) Vascular density (VD) and vascular survival ability (VSAt2/t1 and VSAt3/t1) in the tumor invading front (t1) and inner tumor areas (t2 and t3), as assessed with anti-CD31 moAb (**f**) Linear regression graph of TIL density in the invading tumor front vs. vascular survival ability (VSAt2/t1); (**g**) CD31 immunostaining of a lip carcinoma tissue section with high VD in the invading tumor front (t1-area) (arrows); and (**h**) t2 are of the same tumor showing sustained, still reduced, VD (arrows). All immunohistochemical images are at ×20 magnification.

Cytoplasmic HIF1α expression ranged from 0–98% (median 47), while nuclear expression ranged from 0–27% (median 0%) of cancer cells. High HIF1α expression was noted in 30/60 lip carcinomas, and was significantly linked with low TIL density in the invading tumor front (*p* = 0.02). No other association was noted; Figure 2a,b shows a typical immunohistochemical image of a lip carcinoma with high nuclear and cytoplasmic HIF1α expression.

High LDH5 expression was noted in 40/60 lip carcinomas. High LDH5 expression was significantly linked with low TIL density in the invading tumor front (*p* = 0.04). No other association was noted. Figure 2c,d shows a typical immunohistochemical image of a lip carcinoma with high nuclear and cytoplasmic LDH5 expression.

Strong cytoplasmic VEGF expression was noted in 38/60 cases (range of positive cells 10–80%); Supplemental Figure S1. Linear regression analysis showed a direct association of VEGF expression with LDH5 expression (*p* = 0.009, r = 0.33), however, there was no relation with TIL and TLS densities.

### 3.4. VD and VSA vs. TILs/TLSs

The vascular density VD in the invading tumor front ranged from 42–217 microvessels per ×20 optical field (median 138). The median VD dropped to 52 and 50 in t2 and t3 inner tumor areas; *p* < 0.0001; Figure 2e. Using the ratio of VD t2/t1 and t3/t1 we calculated the vascular survival ability (VSA) of each tumor. This index shows the ability of vessels to survive in inner tumor area. This ranged from 0.005–1 for t2/t1 and from 0.01–0.83 for t3/t1. Linear regression analysis of the above angiogenesis-related variables with the TIL and TLS densities showed a significant inverse association between TIL density in the invading tumor front and VSAt2/t1 (*p* = 0.009, r = 0.33). Figure 2f,g shows CD31 immunostaining of a lip carcinoma tissue section with high VD in the invading tumor front (t1 area), and Figure 2h shows evident reduction, though still high VD in t2 area.

### 3.5. p16, MIB1 vs. TILs/TLSs

Twenty-eight out of sixty lip carcinomas showed p16 reactivity, although only in six between 3–6% and one with 50% reactivity (the rest had 1% reactivity). There was no association of p16 expression with TIL and TLS density, although a marginally higher VD (*p* = 0.07) and VSAt2 (*p* = 0.08) in inner tumor areas was recorded in p16^+^ cases (Supplemental Figure S2).

The MIB1 proliferation index ranged from 3–79% of cancer cell nuclei (median 31). Using this value, cases were grouped into low and high MIB1 index. There was no association with TIL and TLS density (Supplemental Figure S2).

### 3.6. TIL Subtype Analysis

**Figure 3:**
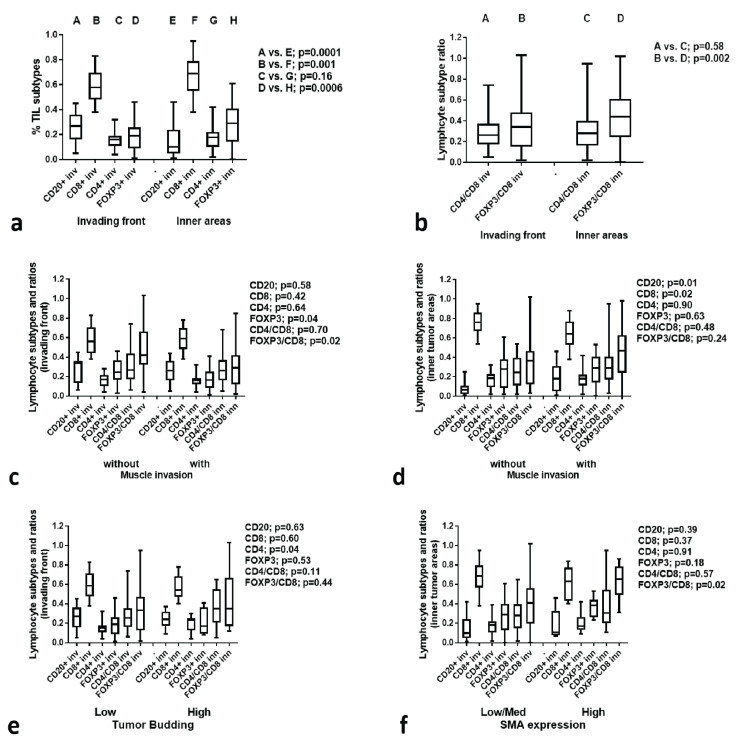
TIL subtypes and ratios: (**a**) TIL subtype composition in the invading front and inner tumor areas; (**b**) TIL subtype ratio in the invading front and inner tumor areas; (**c**) TIL subtypes and ratios in the invading front according to the muscle invasion; (**d**) TIL subtypes and ratios in inner areas according to the muscle invasion; (**e**) TIL subtypes and ratios according to the tumor budding in the invading front (**f**) TIL subtypes and ratios according to the SMA expression in the stroma of inner tumor areas.

Figure 3a shows the fraction of CD20^+^, CD8^+^, CD4^+^, and FOXP3^+^ TILs in the invading tumor front and in inner tumor areas of lip carcinomas. The fraction of CD20^+^ TILs was significantly lower in the inner tumor areas (*p* < 0.0001), while the CD8^+^ one was higher (*p* = 0.001). FOXP3^+^ TILs were significantly more abundant in the inner tumor areas (*p* = 0.0006). We further calculated the CD4^+^/CD8^+^ and FOXP3^+^/CD8^+^ (regulatory/effector) ratios. The FOXP3^+^/CD8^+^ ratio increased significantly in the inner tumor areas (*p* = 0.002) (Figure 3b).

### 3.7. TIL Subtypes vs. Histological Variables

Analysis of CD20^+^, CD8^+^, CD4^+^, FOXP3^+^ TIL subtypes and CD4^+^/CD8^+^, FOXP3^+^/CD8^+^ ratios (whether in the invading front or inner areas) showed no significant association with tumor size.

Analysis in the invading tumor front between the groups of lip tumors without muscle invasion vs. with muscle invasion showed a significant increase of CD20^+^ TILs and a decrease of CD8^+^ TILs in muscle-invading tumors (*p* = 0.01 and 0.02, respectively) (Figure 3c). In inner tumor areas, FOXP3^+^ TILs and FOXP3/CD8 ratio were significantly higher in tumors invading the muscle (*p* = 0.04 and 0.02, respectively) in inner tumor areas (Figure 3d).

Analysis between the groups of lip tumors according to the low and high presence of tumor budding showed a significant increase of CD4^+^ TILs in the invading front in the high TB group (*p* = 0.04) (Figure 3e). No association of TB with CD parameters was found in inner tumor areas. Analysis between the groups of lip tumors with low/moderate vs. high expression of SMA showed a significant increase of the FOXP3^+^/CD8^+^ ratio in high SMA tumors (*p* = 0.02) (Figure 3f)**.**

### 3.8. TIL Subtypes vs. Immunohistochemical Variables

**Figure 4:**
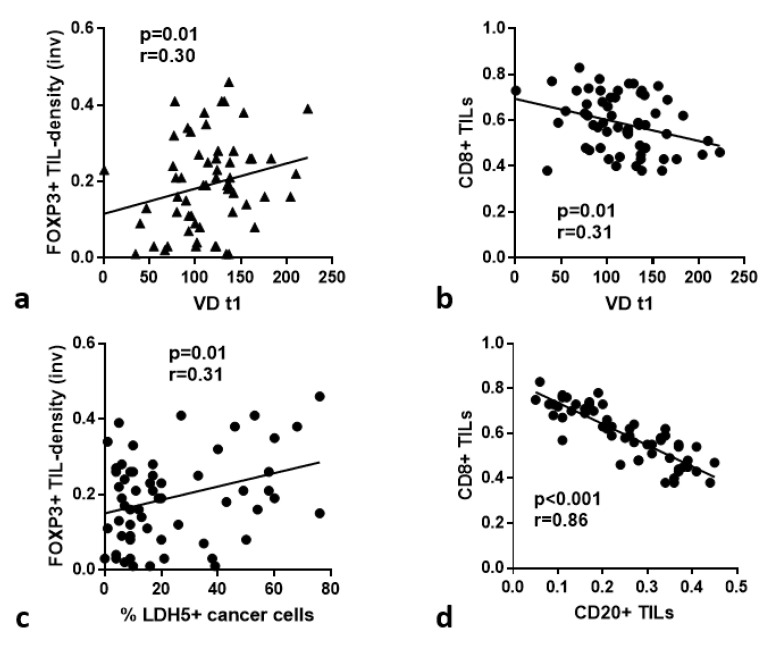
Linear regression analysis of lymphocyte subtype density and immunohistochemical variables: (**a**) FOXP3^+^ TILs vs. VD in the t1 area; (**b**) CD8^+^ TILs vs. VD in the t1 area; (**c**) FOXP3^+^ TIL density in the invading tumor front vs. LDH5 cancer cell expression; (**d**) CD20^+^ TILs vs. CD8^+^ TILs.

Using linear regression analysis, we assessed the association of CD20^+^, CD8^+^, CD4^+^, FOXP3^+^ TIL-subtypes and CD4^+^/CD8^+^, FOXP3^+^/CD8^+^ ratios with the expression of HIF1α, LDH5, VD (t1, t2 and t3) and VSA (t2 and t3) in the invading tumor front. A direct association of VD t1 with CD4^+^ and FOXP3^+^ TILs in the invading tumor front was noted (*p* = 0.05 and 0.01) (Figure 4a). Moreover, VDt1 was inversely related to CD8^+^ TILs in the invading tumor front (*p* = 0.01, r = 0.31) (Figure 4b). LDH5 was directly related to high CD4^+^ TILs in the invading tumor front (*p* = 0.05, r = 0.26), while nuclear LDH5 expression was directly linked with FOXP3^+^ TILs (*p* = 0.01, r = 0.31; Figure 4c) and FOXP3/CD8 ratio (*p* = 0.03, r = 0.27). CD20^+^ TILs were strongly and inversely related to CD8^+^ TILs (*p* = 0.001, r = 0.86) (Figure 4d).

### 3.9. CD68^+^ Macrophage Analysis

The stroma area covered by CD68^+^ macrophages ranged from 0–30% in the invading front and inner tumor areas, however, the median % was significantly lower in inner tumor areas (10% vs. 20%, *p* = 0.001). High CD68^+^ macrophage infiltration in the invading front and inner areas was significantly related to higher rates of muscle invasion (median CD68 score 20% vs. 10%, *p* = 0.006, and 10% vs. 7.5%, 0.01, respectively). There was no association with size, tumor budding, SMA and p16 expression or MIB1 proliferation index.

There was no association between the extent of CD68^+^ staining with TIL or TLS density. Linear regression analysis showed a strong inverse association with HIF1α expression in both the invading front and inner areas (*p* = 0.0005, r = 0.44, and *p* = 0.008, r = 0.33, respectively). Moreover, high CD68^+^ macrophage infiltration was related to high VD t1 and t2 in the invading front (*p* = 0.03, r = 0.28 and 0.003, r = 0.36, respectively) and high VSA t2 (*p* = 0.04, r = 0.26). 

## 4. Discussion

TME has remained in the research spotlight over recent decades. Studies focus on the continuous interaction between cancer cells, cellular components of the tumor stroma, and the physio-chemical conditions prevailing in the TME that guide the tumor growth, invasion, and metastasis [29]. Immune cells infiltrating TME have the ability to turn against cancer cells inhibiting tumor spread. Nevertheless, under certain conditions, they differentiate into immunosuppressive immune cell populations that lead to immune escape. Concerning immune cells of the TME and their impact on tumor growth and metastasis (and consequently prognosis), lymphocytes are the best studied. The presence of these cells in the tumor microenvironment, either in the form of a diffuse infiltration or as aggregates of lymphocytes (with or without a germinal center), called tertiary lymphoid structures (TLSs), has been shown to have a good prognosis in solid cancers, including head and neck carcinoma [24,30,31,32,33,34].

In the present study, we examined the TME of lip SCC, using immunohistochemical markers of hypoxia, metabolism and angiogenesis, and how these affect the immune response in terms of TIL density and TLS formation, as well as in terms of the prevalence of cytotoxic and regulatory T-cells. Authors have proposed many ways to evaluate the density of TILs, while many others follow the instructions proposed by the International TILs Working Group (ITWG) in 2014. In brief, guidelines suggest the calculation of lymphocytic infiltration as the percentage of stromal TILs in the whole area of stroma tissue, when artifacts, necrotic areas, and polymorphonuclear cells were excluded from the assessment [35]. Three years later, Hendry et al. adjusted the guidelines published by ITWG so that they can also be applied to solid tumors in general, and not only for breast carcinomas [36]. Regarding tertiary lymphoid structures, there is not a standard way of evaluation. Some authors use immunohistochemistry to highlight them, however, official recommendations are not yet proposed [37,38,39].

In our study, we counted the number of lymphocytes in the invading front of the tumor and inside the tumor stroma in ×400 magnification in all available optical fields, and the total number of lymphocytes was divided by the total amount of high-power optical fields to score each case. According to the median value, cases were categorized as high and low TIL density. This method, although laborious and time-consuming, overcomes the subjectivity of each pathologist in the evaluation of lymphocytic infiltration and increases reproducibility. Using an analogous approach to assess TLS density, we calculated the number of all TLSs in the whole slide at high-power (×400) magnification and then divided it by the total number of optical fields. Tumors were categorized as low and high TLS density. We considered a relatively circumscribed accumulation of lymphocytes as TLS, whether it showed a germinal center or not, without the use of immunohistochemistry.

Compared to other head and neck carcinomas (particularly laryngeal SCC and SCC of the tongue), we found that, in inner tumor areas, TIL density was significantly higher in lip carcinomas. Caruntu et al. and Zancope et al. showed that CD8^+^ lymphocytic density was higher in lip cancer [40,41]. On the other hand, Green et al. reported that TIL levels are higher in oropharyngeal SCC [42], while others found no relation at all [43,44].

Regarding tumor size and its relation with TIL and TLS density, statistically significant lower TIL values were noted in larger tumors, both in the invading front and inner areas, a result that is in agreement with what other authors have reported about head and neck carcinoma [31,43,45,46]. Moreover, TIL density in the invading front was significantly lower in tumors with deep invasion, an association described in other studies in oral SCC, gastric adenocarcinoma and melanoma [43,47,48].

We further focused on the association of other histological and immunohistochemical variables with immune response. Tumor budding is continuously gaining ground, not only in research but also in daily practice. However, there was no association of TB numbers with TIL or TLS density in our statistical analysis. Lang-Schwarz et al. reached the same results, as they found no correlation among them in a series of colorectal carcinomas [49]. On the contrary, Safaa et al. and Zhang et al. established a negative correlation of lymphocytic tumor infiltration and tumor budding in gastric adenocarcinoma [50,51]. The presence of CAFs was also estimated using SMA immunohistochemical markers, and high SMA expression was linked with low TIL density in the invading tumor front, which is supported by other authors in studies of colorectal cancer [52,53].

Expression of immunohistochemical marker p16 was low in our study. This low expression was expected, as most cases of lip cancer are not HPV-associated. Due to this low expression, no valuable information could be extracted. However, Xu et al. have showed that no association exists between TIL density and p16 in head and neck SCC (specifically, oropharyngeal SCC) [54]. Moreira et al. support a positive link among CD4^+^ lymphocytic levels in the tumoral stroma of oral SCC, while Zancope et al. exhibited an inverse interconnection of peritumoral and intratumoral CD8^+^ lymphocytes in lip cancer and SCC of the oral cavity with the proliferative index of cancer cells [41,44]. In our study we found no association of cancer cell proliferation index with TIL or TLS density.

The tumor microenvironment is often hypoxic and acidic. Hypoxia conditions in the tumor microenvironment are reflected in HIF1α and LDHA expression [55,56]. High HIF1α expression was noted in half of the cases in our report and was significantly linked with low TIL density in the invading tumor front, a statement already supported by Giatromanolaki et al. and Koukourakis et al. in a series of breast and head and neck carcinomas, respectively [24,31]. In the exact same study, Giatromanolaki et al. proposed a negative relation between TIL density in the invading tumor front and LDH5 expression [24], which we confirmed in the present report.

Hypoxia and HIF1α participate in the process of blood vessel formation via direct control of VEGF gene expression, the major director of angiogenesis [57,58]. Using the CD31 marker, we assessed immunohistochemically the microvessels in the invading tumor front (t1) and in two consecutive optical fields (by moving the optical field towards the tumor center; t2, t3 areas). Using this method, we calculated the vascular survival ability (VSA) of the produced tumor vasculature. High angiogenic activity was linked with high CD4^+^, FOXP3^+^, and low CD8^+^ TIL density, as well as high CD68^+^ macrophage presence. This could be a result of angiogenic growth factors secreted by cancer cells or macrophages; we found no association with VEGF expression. Mukherjee et al. and Cho et al. also observed a positive relationship between low CD8^+^ TIL density and larger size of tumors in a series of oral SCCs [59,60], while Green et al., Lequerica-Fernández et al., and Wang et al. agree with our results about the positive relation of FOXP3^+^ lymphocytic density and tumor size [42,61,62].

We also found an association between high presence of tumor-budding and increased CD4^+^ T-cell infiltration in the invading front of the tumor. Jang noticed an interconnection among CD8^+^ TILs of the invading front and inner stroma with tumor budding in colorectal cancer [63]. Moreover, we found a significantly higher FOXP3^+^/CD8^+^ ratio in tumors with high SMA expression, suggesting a role of activated stroma fibroblasts in sustaining regulatory T-cell activity. Of interest, infiltration of the tumor stroma with CD20^+^ B-cells was inversely related to the presence of cytotoxic CD8^+^ T-cells, and directly linked with invasive ability, suggesting that the role of B-cells in the immune landscape of lip cancer deserves further evaluation.

## 5. Conclusions

In the current study, we highlight the importance of existing conditions in the TME and the way that they affect the immune response. Lip SCCs appeared as more immunogenic compared to other HNCs. Poor lymphocytic infiltration was related to larger size of tumors, ability of tumors to invade deeper in the tissue, dense presence of activated stroma fibroblasts and hypoxic conditions. Low CD8^+^ TIL density and high FOXP3^+^/CD8^+^ ratio characterize larger tumors, intense proliferative activity and high angiogenic activity. This study provides insights into the interactions between TME and immune response, which may be useful in the development of immunotherapy strategies targeting TME.

## Data Availability

All data, tissue material, stained slides, and analysis have been generated in the University Hospital of Alexandroupolis and are available upon reasonable request.

## Supplementary Materials

The following supporting information can be downloaded at: https://www.mdpi.com/article/10.3390/cancers15051478/s1. Figure S1. Typical immunohistochemical images (arrows show staining): (a) SMA staining of the tumor stroma fibroblasts (arrows); (b) Nuclear staining of MIB1 in cancer cells; (c) Cytoplasmic and nuclear staining of p16 in cancer cells; (d) Membrane CD4 staining in TILs; (e) Membrane CD8 staining of TILs; (f) Nuclear staining of FOXP3 in TILs; (g) Membrane CD20 staining in TILs; (h) CD68 staining by stroma infiltrating macrophages; (i) VEGF staining of cancer cells. All images were captured at ×20 magnification. Figure S2. TIL density and TLS density in the invading tumor front and inner tumor areas according to: (a) p16 status; (b) MIB1 proliferation index.
