# Supplementary material for: Tumor Microenvironment and Immune Response in Lip Cancer
Source: Cancers (Basel). 2023 Feb 25;15(5):1478. doi: 10.3390/cancers15051478 (PMC10001350; doi:10.3390/cancers15051478)
Supplement: Supplementary file 1 [file cancers-15-01478-s001.zip › cancers-2215250-supplementary.pdf]

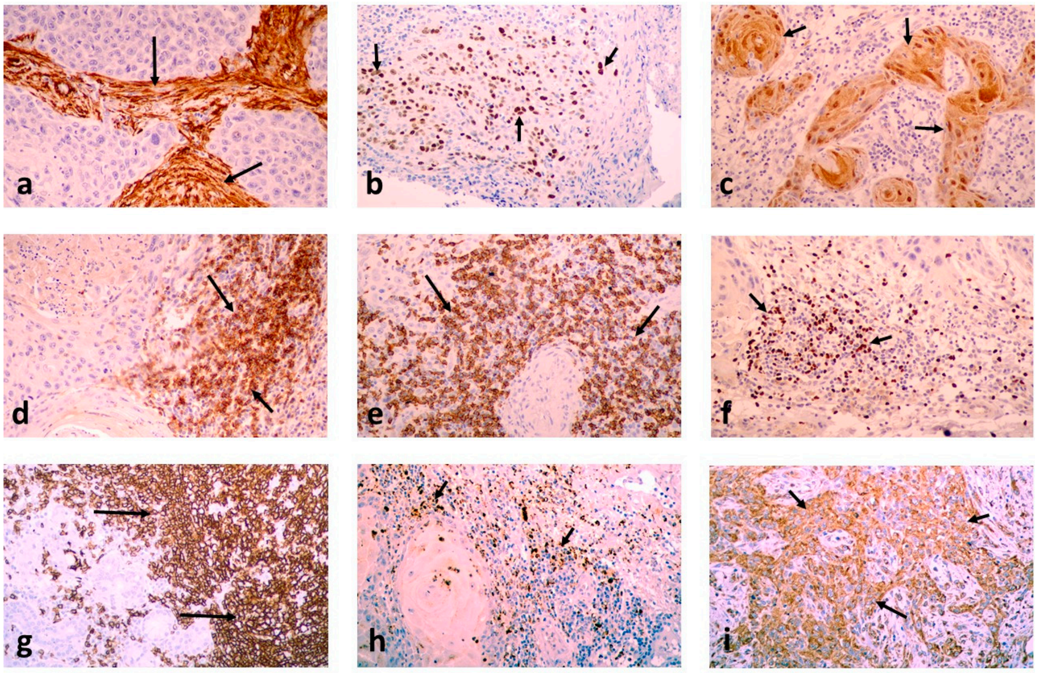

Figure S1. Typical immunohistochemical images (arrows show staining): (**a**) SMA staining of the tumor stroma fibroblasts (arrows); (**b**) Nuclear staining of MIB1 in cancer cells; (**c**) Cytoplasmic and nuclear staining of p16 in cancer cells; (**d**) Membrane CD4 staining in TILs; (**e**) Membrane CD8 staining of TILs; (**f**) Nuclear staining of FOXP3 in TILs; (**g**) Membrane CD20 staining in TILs; (**h**) CD68 staining by stroma infiltrating macrophages; (**i**) VEGF staining of cancer cells. All images were captured at ×20 magnification.

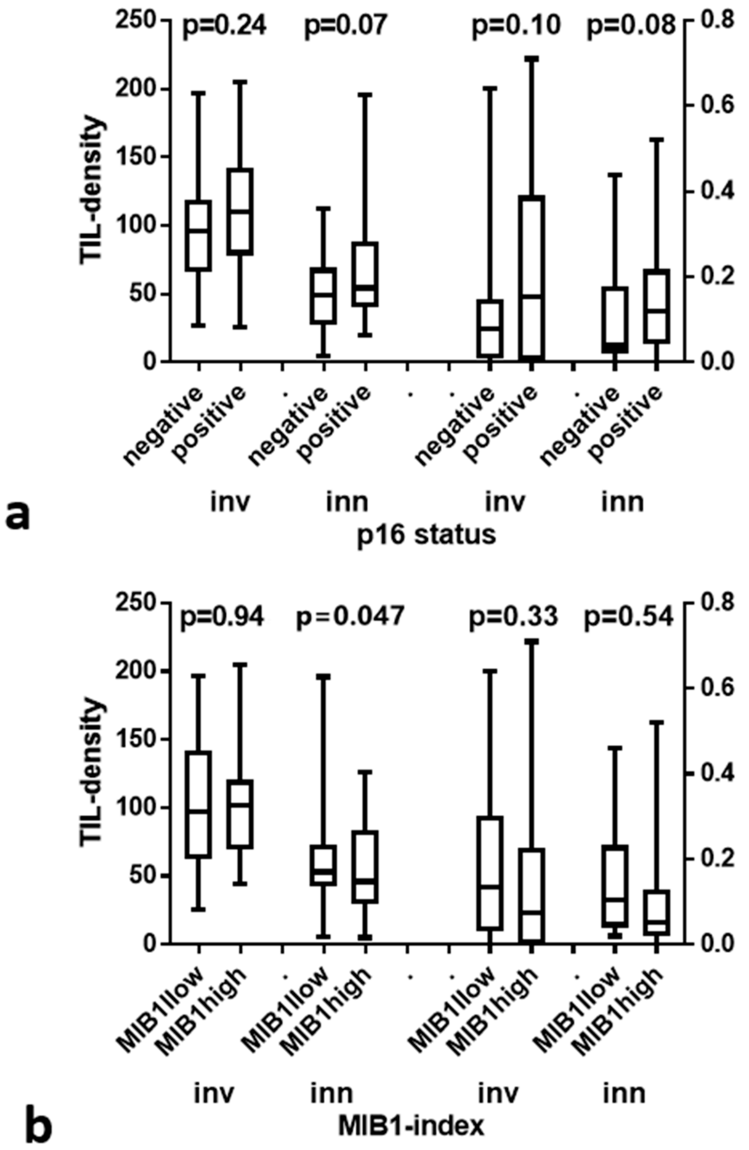

Figure S2. TIL density and TLS density in the invading tumor front and inner tumor areas according to: (**a**) p16 status; (**b**) MIB1 proliferation index.
